# Impact of cervical sagittal parameters on axial neck pain in patients with cervical kyphosis

**DOI:** 10.1186/s13018-020-01909-x

**Published:** 2020-09-22

**Authors:** Jia Li, Di Zhang, Yong Shen

**Affiliations:** 1 Department of Orthopedic Surgery, The Third Hospital of Hebei Medical University, 139 Ziqiang Road, Shijiazhuang, 050051 P. R. China; 2 The Key Laboratory of Orthopedic Biomechanics of Hebei Province, The Third Hospital of Hebei Medical University, 139 Ziqiang Road, Shijiazhuang, 050051 P. R. China

**Keywords:** Cervical kyphosis, Axial neck pain, Cervical sagittal alignment, T1 slope, C2-7 sagittal vertical axis

## Abstract

**Background:**

Cervical sagittal alignment (CSA) is closely related with cervical disk degeneration and impacts the spinal function, especially in the setting of cervical kyphosis (CK). In this study, we evaluated the influence of cervical sagittal parameters on the development of axial neck pain (ANP) in patients with CK.

**Methods:**

Data pertaining to 263 patients with CK who visited the outpatient department of our hospital between January 2012 and December 2018 were retrospective analyzed. The most common symptoms of ANP were neck pain, stiffness, or dullness. Visual analog scale (VAS) was used to evaluate ANP. The following radiographic parameters were evaluated: CK types, C2-7 sagittal vertical axis (SVA), thoracic inlet angle (TIA), T1 slope, neck tilt (NT), cranial tilt, and cervical tilt. Sagittal alignment of CK was classified into 2 types: global and regional type. Multivariate logistic regression analysis was performed to identify risk factors for ANP.

**Results:**

Patients who complained of ANP were categorized as ANP group (VAS score ≥ 3; *n* = 92), while those without ANP were categorized as non-ANP group (VAS score < 3; *n* = 171). There was no significant between-group difference with respect to age (*P* = 0.196), gender (*P* = 0.516), TIA (*P* = 0.139), NT (*P* = 0.676), CK type (*P* = 0.533), cranial tilt (*P* = 0.332), cervical tilt (*P* = 0.585), or cervical disk degeneration (*P* = 0.695). The T1 slope and C2-7 SVA in the ANP group were significantly greater than that in the non-ANP group (*P* < 0.05). On multivariate logistic regression, C2-7 SVA [

odds ratio (OR) 2.318, 95% confidence interval 1.373–4.651, *P* = 0.003) and T1 slope (OR 2.563, 95% CI 1.186–4.669, *P* = 0.028) were identified as risk factors for ANP.

**Conclusions:**

Our findings suggest a significant effect of cervical sagittal parameters on the occurrence of ANP in patients with CK. Greater T1 slope and larger C2-7 SVA may lead to the development of neck pain.

## Background

Sagittal alignment of the cervical spine is affected by multiple factors. It is important to identify the risk factors for degeneration of the cervical spine. Cervical sagittal imbalance is the one of the main reasons for cervical disk degeneration and associated disorders [1, 2]. Misalignment exacerbates the load on the intervertebral disk and posterior joints, which accelerates the progression of spinal degenerative diseases. Patients with cervical sagittal imbalance are more likely to develop axial neck pain (ANP). The relationship between cervical sagittal alignment (CSA) and axial neck pain (ANP) is not well characterized in contemporary literature. Moreover, it is difficult to evaluate if the patients are classified into proper subgroups. In previous studies, the Cobb angle method was typically used for assessment of CSA. However, this method does not allow for precise assessment of the segmental deformities [3–6]. Owing to the segmental deformities, results of Cobb angle method for assessment of cervical alignment may be misleading, especially in patients with cervical kyphosis (CK).

Thoracic inlet angle (TIA) is considered by many researchers to be as important as pelvic incidence (PI). In particular, the T1 slope is considered as a crucial parameter for the determination of CSA. However, as a result of shoulder and thoracic trunk, T1 slope may not be accurately assessed sometimes [7–9]. To overcome these limitations, magnetic resonance imaging (MRI) has been used for measuring these parameters and for evaluating spinal degeneration. In a study by Oshina et al., the use of standing radiographs and supine MRI for assessment of sagittal alignment yielded similar results in patients with CK [10].

The causation of ANP is widely considered to be multifactorial. However, to the best of our knowledge, the relationship between cervical sagittal parameters and occurrence of ANP is not well characterized, especially in the context of CK. We hypothesized that patients with different CK types who have different potential abilities to develop ANP. The purpose of this study was to evaluate the influence of cervical sagittal parameters on ANP in patients with CK.

## Methods

Data pertaining to 263 patients with CK who visited the outpatient department of our hospital between January 2012 and December 2018, were retrospectively analyzed. All patients had undergone MRI and radiograph of the cervical spine. Patients with ANP most commonly complained of neck pain, stiffness, or dullness. The exclusion criteria were patients with tumor, spinal infection, rheumatic disease, cervical fractures, history of cervical spine surgery, and traumatic injuries. Patients for whom detailed radiographic parameters could not be obtained were also excluded. Visual analog scale (VAS) was used to evaluate ANP. Patients with ANP VAS scores ≥ 3 were categorized as ANP group; patients with ANP VAS scores < 3 were categorized as non-ANP group. This study was approved by the Ethics Committee of the Third Hospital of Hebei Medical University, China. The requirement for informed consent of patients was waived off as all data were anonymized prior to processing and analysis. All methods were conducted in accordance with the approved guidelines.

### Radiographic evaluation

**Fig. 1:**
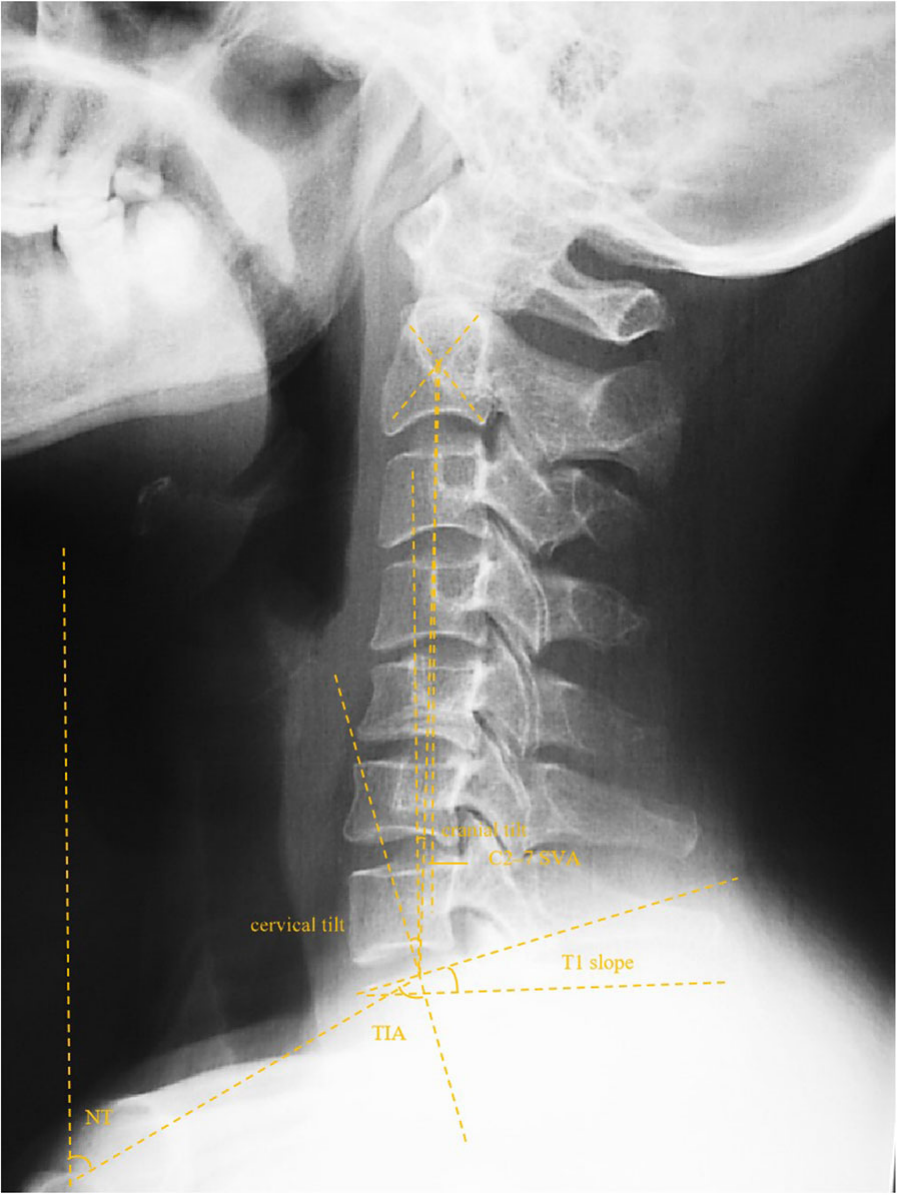
Schematic illustration showing the methodology for measurement of radiographic parameters. T1 slope, the angle between a horizontal line and the upper end plate of T1. NT, the angle formed by a vertical line from the sternum tip and a line connecting the center of the T1 upper end plate and the upper end of the sternum. TIA, the angle formed by a perpendicular line off the T1 upper end plate and another line connecting the center of the T1 upper end plate and the upper point of the sternum. C2-7 SVA, the distance from the posterosuperior corner of C7 to a vertical line from the center of the C2 vertebra. Cranial tilt, angle between the vertical line from the upper end plate of T1 and a line from the center of the upper end plate of T1 to the C2 center. Cervical tilt, angle between the two lines originating from the center of the T1 upper end plate: the vertical line and the line from the center of upper end plate of T1 to the C2 center

**Fig. 2:**
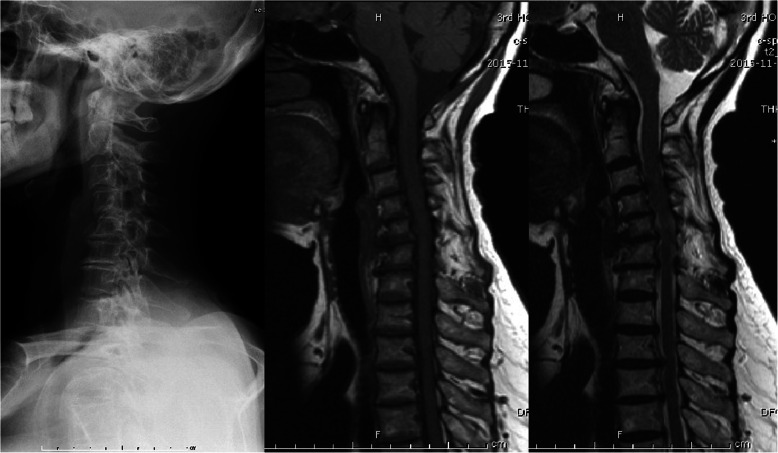
Global type, all the centroids are posterior to the C2–C7 centroid line and the distance between at least 1 centroid and the line is ≥ 2 mm

**Fig. 3:**
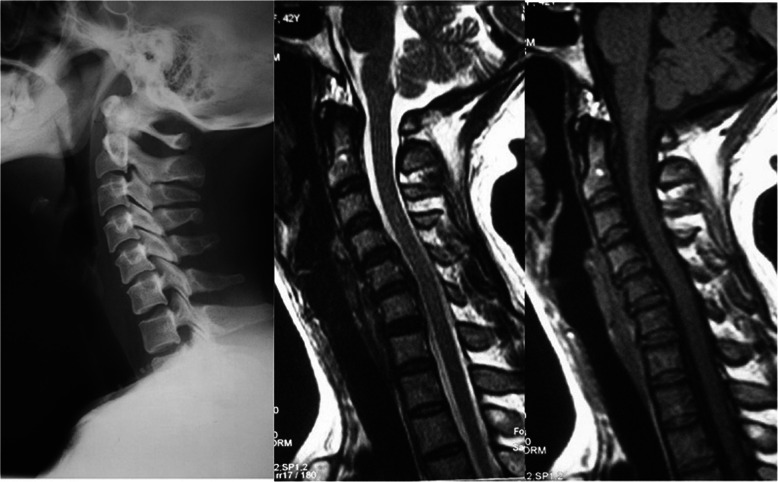
Regional type I, at least 1 of the upper cervical centroids is anterior to and at least 1 of the lower cervical centroids is posterior to the C2–C7 centroid line; in addition, the distance between the C2–C7 centroid line and at least 1 centroid is ≥ 2 mm

**Fig. 4:**
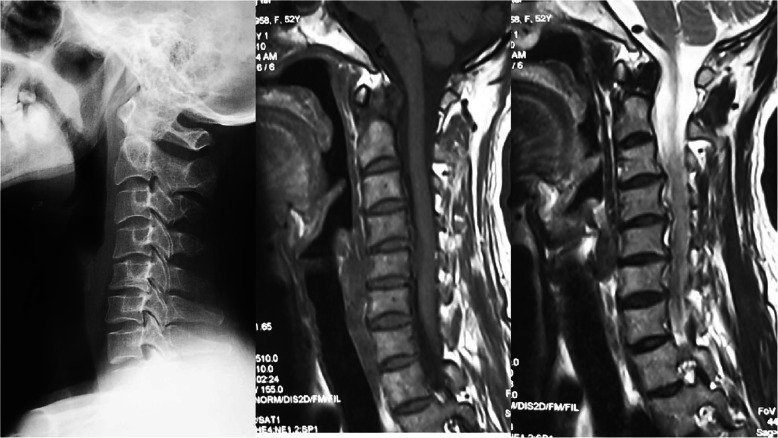
Regional type II, at least 1 of the upper cervical centroids is posterior to and at least 1 of the lower cervical centroids is anterior to the C2–C7 centroid line; in addition, the distance between the C2–C7 centroid line and at least 1 centroid is ≥ 2 mm

Radiographic evaluation was performed at the authors’ department. All radiographs were analyzed by two doctors who were blinded to clinical information. The mean values were applied for analysis. Cervical disk degeneration was assessed based on signal intensity and/or decrease in the height of disk, with/without posterior disk protrusion. The following cervical sagittal parameters were measured: T1 slope was measured as the angle between a horizontal line and the upper-end plate of T1. Neck tilt (NT) was measured as the angle between a vertical line from the sternum tip and a line connecting the center of the T1 upper-end plate and the upper end of the sternum. Thoracic inlet angle (TIA) was measured as the angle between a perpendicular line off the T1 upper-end plate and another line connecting the center of the T1 upper-end plate and the upper point of the sternum (T1 slope + NT). C2-7 sagittal vertical axis (SVA) was measured as the distance from the posterosuperior corner of C7 to a vertical line from the C2 center. Cranial tilt was measured as the angle between the vertical line from the upper-end plate of T1 and a line from the center of the upper-end plate of T1 to the C2 center. Cervical tilt was measured as the angle between the two lines originating from the center of the T1 upper-end plate: the vertical line and the line from the center of upper-end plate of T1 to the C2 center (Fig. 1). CK was classified as either global type (all of the vertebral centroids are posterior to the C2-7 vertebral centroids line) (Fig. 2) or regional type (one of the upper vertebral centroids was anterior to and one of the lower vertebral centroids was posterior to the C2-7 vertebral centroids line, or one of the upper vertebral centroids was posterior to and one of the lower vertebral centroids was anterior to the C2-7 vertebral centroid line (Figs. 3 and 4).

### Statistical analysis

Statistical analysis was performed using the SPSS software (version 22.0, Chicago, IL, USA). Continuous variables are shown as mean ± standard deviation. Continuous variables were analyzed using *t* test, while categorical variables were analyzed using the chi-squared test. Multivariate logistic regression analysis was performed to identify risk factors for ANP. For all analyses, *p* values < 0.05 were considered indicative of statistical significance.

## Results

The patient characteristics and cervical sagittal parameters in the ANP and non-ANP groups are summarized in Table 1. A total of 263 patients (120 males and 143 females) with CK were included in the study. Of these, 92 patients (35.0%) complained of ANP (ANP group), while 171 patients (65.0%) did not complain of ANP (non-ANP group). There were no significant differences between the two groups with respect to age (*P* = 0.196), gender (*P* = 0.516), TIA (*P* = 0.139), NT (*P* = 0.676), CK type (*P* = 0.533), cranial tilt (*P* = 0.332), cervical tilt (*P* = 0.585), or cervical disk degeneration (*P* = 0.695). However, the T1 slope in the ANP group (26.3° ± 6.2°) was significantly greater than that in the non-ANP group (21.5° ± 7.6°; *P* = 0.027). C2-7 SVA in the ANP group (20.9 ± 10.3 mm) was significantly larger than that in the non-ANP group (*P* = 0.003).

**Table 1:** Comparison of demographic characteristics and radiological parameters between the ANP group and non-ANP group

	ANP group	Non-ANP group	*P* value
Age (years)	43.5 ± 12.9	40.8 ± 13.6	0.196
Gender (M/F)	39/53	81/90	0.516
CK type			0.533
Global type	61	77	
Regional type	50	75	
Cervical disk degeneration			0.695
Yes	39	68	
No	53	103	
C2-7 SVA (mm)	20.9 ± 10.3	13.3 ± 7.5	0.003
T1 slope (°)	26.3 ± 6.2	21.5 ± 7.6	0.027
NT (°)	47.1 ± 5.1	49.6 ± 6.8	0.676
TIA (°)	73.5 ± 5.6	70.2 ± 7.0	0.139
Cranial tilt (°)	5.2 ± 6.5	4.91 ± 7.11	0.332
Cervical tilt (°)	17.3 ± 15.3	18.7 ± 16.3	0.585

Using ANP as a dependent variable, a multivariate logistic regression model was used to identify the risk factors. Variables associated with a *P* value < 0.2 in the univariate analysis (age, TIA, T1 slope, and C2-7 SVA) were included in the model as dependent variables by a forward stepwise method. C2-7 SVA [odds ratio (OR) 2.318, 95% confidence interval (CI) 1.373–4.651, *P* = 0.003] and T1 slope (OR 2.563, 95% CI 1.186–4.669, *P* = 0.028) were found to be independent predictors of ANP (Table 2).

**Table 2:** Results of multiple logistic regression analysis showing risk factors for cervical disk degeneration

Risk factor	*P*	Odds ratio	95% CI
Age (years)	0.175	1.539	0.963–2.661
C2–7 SVA (mm)	0.003	2.318	1.373–4.651
T1 slope (°)	0.028	2.563	1.186–4.669
TIA (°)	0.221	1.373	0.834–2.259

## Discussion

Previous studies have shown that CSA plays a key role in the causation of neck pain and associated functional disability. Imbalance of the cervical spine often leads to serious degenerative disease. These studies have verified the relationship between CSA and activities of daily living in asymptomatic patients with or without cervical deformity [11–13]. It is generally acknowledged that ideal cervical balance necessitates minimal muscular energy expenditure and reduces the ANP in daily life. Regardless of lordosis or kyphosis, CSA is important for maintaining global sagittal balance and preventing ANP. Sagittal imbalance is more liable to lead to cervical disk degeneration compared with normal sagittal alignment [14–16]. Cervical sagittal imbalance necessitates excessive energy consumption to achieve body balance and mobility. Over a period of time, the cervical sagittal imbalance leads to ANP and spinal diseases such as disk degeneration and spondylolisthesis. Therefore, cervical sagittal imbalance is considered associated with poor quality of life. This study highlighted the relationship between ANP and CSA and identified C2-7 SVA and T1 slope as independent predictors of ANP in patients with CK.

In previous studies, thoracic inlet parameters were found to exhibit a strong correlation with the other cervical parameters [17–19]. Therefore, assessing the impact of these parameters on ANP in the context of CK is a key imperative. Similar to PI, TIA is considered as a constant morphologic parameter. NT was found to be a constant parameter; a higher T1 slope implies a larger TIA. In a study by Sun et al., patients with sagittal imbalance were found to be at a higher risk of degenerative cervical spondylotic myelopathy. When T1 slope was less than 18.5°, it showed significant diagnostic value for the occurrence of degenerative cervical disease. However, most patients in their study had cervical lordosis, while those with CK were not included [11]. Jouibari et al. compared cervical sagittal parameters between patients with neck pain and healthy controls. In their study, the T1 slope was significantly lower in the neck pain group compared to the healthy control group; however, there was no difference in cervical lordosis between the two groups [12]. Lin et al. investigated 90 patients who underwent cervical surgery; they found that larger C2-7 SVA, lower TIA, and higher NT values were independent predictors of high preoperative neck disability [13]. In the present study, the T1 slope in the ANP group was significantly greater than that in the non-ANP group. On multivariate logistic regression analysis, the T1 slope was a risk factor for ANP. This helps elucidate the occurrence of ANP. In patients with higher T1 slope, if the center of gravity of the head moves forward, it would aggravate the kyphosis and cause ANP. Our findings are similar to those of Le Huec et al. They analyzed radiographic parameters of 106 asymptomatic participants to evaluate sagittal balance and identified CK in almost one-third of participants [20]. This indicates that maintenance of cervical sagittal balance in patients with CK may help prevent neck pain. In other words, CK is a normal physiological state in the presence of cervical balance.

The T1 slope is the only value that links both the cervical and thoracic spine. It shows a close correlation with thoracic kyphosis, TIA, and C2-7 SVA [21]. C2-7SVA is believed to be another important indicator of cervical sagittal balance [22, 23]. The threshold for cervical imbalance is C2-7SVA ≥ 40 mm [24, 25]. In the present study, the C2-7SVA in both groups was < 40 mm, which implied that the cervical spine was in basic equilibrium. It was insufficient to assess the cervical alignment parameters using C2-7SVA and cervical curvature alone. Previous studies have also shown that the T1 slope represents the angle, while C2-7SVA represents global sagittal alignment. Hyun et al. and Tang et al. considered that C2-7 SVA is the best indicator of cervical malalignment, which is significantly impacted by the T1 slope and cervical curvature [23, 26].

Higher thoracic kyphosis often results in a greater T1 slope [23]. It inevitably leads to a compensatory increase in cervical lordosis. However, in patients with CK, this phenomenon is completely different. Staub et al. reviewed the relationship between T1 slope and cervical lordosis in 103 adult patients with spinal deformity. They found that the T1 slope minus cervical lordosis ranging from 14.5° to 26.5° could maintain the horizontal balance [21]. For this reason, C2-7 SVA should be within the normal range if cervical lordosis is high or the T1 slope is low. The worst mismatch is a higher T1 slope and lower cervical lordosis. Compared to cervical lordosis, the relation between the cervical parameters in the context of CK is different. When a smaller C2-7 SVA accompanies a lower T1 slope, it is easier to maintain cervical sagittal balance by the compensatory mechanism of the posterior neck muscles. It helps maintain the center of the head position back to the spinal axis. Otherwise, a larger C2-7 SVA with a higher T1 slope leads to cervical malalignment, which is not compensated by the posterior neck muscles and eventually causes ANP. The translational mobility of upper and middle cervical levels in regional CK type is greater than that in global CK type; a larger C2-7 SVA and higher T1 slope may accelerate disk degeneration at the transition zone. Furthermore, the transition zone and apex of the level are at a higher risk of causing ANP, especially in the setting of larger C2-7 SVA and/or greater T1 slope. For the global CK type, the angular motion is greater at the apex of kyphosis; therefore, a larger C2-7 SVA or a higher T1 may be the reason for ANP. From the evidence available so far, a smaller C2-7 SVA accompanied with a lower T1 slope was tolerated by patients with CK. However, a larger C2-7 SVA with a higher T1 slope may lead to ANP. This explains the identification of larger C2-7 SVA and higher T1 slope as independent predictors of ANP in patients with CK.

With regard to the kinematics of CK, the site of spinal cord compression in the context of regional CK type is at the transition zone while that in the global CK type is at the apex of kyphosis. Extension segmental motion of the global CK type is increased in the upper cervical spine compared to the regional CK type whose position is lordosis. The opposite phenomenon is observed when the cervical spine is in flexion. In spite of the different abnormal kinematics, this result showed that the CK type is not a risk factor for ANP. In previous studies, approximately 20% asymptomatic individuals showed signs of cervical disk degeneration [27–29]. Consistent with these findings, we also observed signs of disk degeneration in the non-ANP group.

In addition, the posterior neck muscles also play an important role in maintaining cervical curvature. For the purpose of minimizing the energy expenditure, patients with CK require strengthening of the posterior paraspinal neck muscles, especially patients with a greater T1 slope. In the ANP group, the mean T1 slope and C2-7 SVA were significantly greater than that in the non-ANP group. Theoretically, a higher T1 slope and larger C2-7 SVA may increase the risk of cervical sagittal imbalance in patients with CK; this may increase the fatigue of posterior paraspinal neck muscles and eventually cause neck pain.

### Limitations

Some limitations of our study should be considered while interpreting the results. Firstly, there was a lack of global spinal sagittal radiographs to estimate the mutual effect of the lumbar and thoracic spine and CK. Secondly, kinematic MRI is a better method than supine MRI for the assessment of cervical instability and degeneration. Thirdly, owing to the retrospective study design, it was difficult to control for all potential confounding variables. Fourth, the sample size in our study was relatively small. Larger studies with long-term follow-up are required to better characterize the relationship between CK and ANP. Future studies should also explore the correlation between global spinal balance and development of ANP.

## Conclusions

We found a significant effect of cervical sagittal parameters on the occurrence of ANP in patients with CK. Greater T1 slope and larger C2-7 SVA were closely associated with the development of neck pain in this study.

## Data Availability

Not applicable.
